# Conducting Screenings and Determining Primary Healthcare Provider Follow-Up for Heart, Muscle, and Brain Health in Older Adults at a Community Pharmacy: An Implementation-Oriented Pilot Study

**DOI:** 10.20900/agmr20260006

**Published:** 2026-03-23

**Authors:** Jayme Steig, Jacob Kieser, Ryan McGrath

**Affiliations:** 1 Department of Pharmacy Practice, North Dakota State University, Fargo, ND 58102, USA; 2 Department of Health, Nutrition, and Exercise Sciences, North Dakota State University, Fargo, ND 58102, USA

**Keywords:** aging, arrhythmias, dementia, geriatrics, hand strength, referral and consultation

## Abstract

**Background:**

Frequently visited community pharmacies can serve as sites for additional healthcare, including screenings for unknown conditions. Our implementation-oriented pilot study sought to evaluate the proportions of older adults that followed-up with their primary healthcare provider about a positive cardiac arrhythmia, weakness, or cognitive impairment screening performed in a community pharmacy.

**Methods:**

A cross-sectional design was used and screenings occurred at a well-visited community pharmacy inside a clinic. Possible cardiac arrhythmias were identified with a mobile electrocardiogram. Weakness status was examined with a hydraulic handgrip dynamometer. A paper-and-pencil based cognitive assessment determined cognitive impairment status. Participants screening positive were advised to visit their healthcare provider, and follow-up for determining if such visits occurred was performed approximately 3-months later.

**Results:**

Of the 106 participants, 51 were referred to their healthcare provider for any positive screening. At follow-up, 12 (23.5%) participants reported visiting their primary healthcare provider about the positive screening performed at the community pharmacy.

**Conclusions:**

Our implementation-oriented pilot study showed promise for kindling healthcare provider follow-up regarding positive screenings performed at a community pharmacy. Conducting such screenings may reengage patients with their primary healthcare providers when appropriate, and support the identification of unknown conditions.

## INTRODUCTION

Approximately 30 million Americans are living with an undiagnosed disease, and the presence of an undiagnosed condition is associated with multimorbidity, disability, and accelerated time to mortality [1]. While there are many explanations as to why there is a high prevalence of undiagnosed conditions in the United States, nearly a third of Americans are not routinely visiting their healthcare provider [2]. Age is a known risk factor for several health conditions [3], and the older adult population is projected to rapidly increase [4]. Specifically, most older adults are living with multimorbidity [5], and the proportion of older adults with such a disease clustering is estimated to elevate as the older adult population grows [6]. Older adults also lack routine visitation to their healthcare provider, which may exacerbate the progression of an unknown health condition [7]. Therefore, reaching older adults for reestablishing routine healthcare engagement is crucial for mitigating the health implications of diagnosed and undiagnosed diseases.

Community pharmacists are well-positioned to improve healthcare access, as patients visit their pharmacy nearly twice as often relative to their primary healthcare provider [8]. This consistency in visitation enables opportunities for pharmacists and related personnel to conduct patient screening, and encourages additional healthcare as appropriate [8]. For example, community pharmacists-based programs have improved diabetes, asthma, hypertension, and dyslipidemia management [9-11].

Older adults are at risk for a multitude of health conditions [12], thereby elevating complexity in screening selection. Cardiac arrhythmias are common in older adults [13], which may elevate risk for morbidity and mortality [14]. Muscle mass and strength decrease during aging [15], and handgrip strength is a convenient and reliable assessment of muscle health [16]. Weakness, as measured by handgrip strength, is present in many older Americans [17], and is associated with diabetes, osteoporosis, and functional limitations [18]. Cognitive impairment screening tools are an excellent assessment of brain health that are likewise predictive of dementia [19]. Alzheimer’s disease and Alzheimer’s disease related dementias are a leading cause of death in the United States, and dementia prevalence is expected to elevate substantially alongside the rapidly growing older adult demographic [20]. Given the public health significance of cardiac arrhythmias, weakness, and cognitive impairment, community pharmacists may target these feasible and diverse health indicators for screening in effort to reduce undiagnosed disease, present health-related awareness, and extend healthy aging. This implementation-oriented pilot study sought to examine the proportions of older adults that followed-up with their primary healthcare provider about a positive heart, musculoskeletal, or cognitive screening conducted in a community pharmacy.

## MATERIALS AND METHODS

A cross-sectional design was utilized for this implementation-oriented pilot study. The study occurred at an independently owned and operated community pharmacy located inside a major healthcare clinic that serves over 2000 older adults annually. Adults aged ≥ 65-years that received a prescription at the participating community pharmacy were eligible. Persons diagnosed with cancer, dementia, a neurological condition (e.g., stroke), aphasia, or an inability to perform muscle function testing due to pain, arthritis, or a limiting procedure on both hands were excluded. The North Dakota State University Institutional Review Board approved study protocols, and all participants provided written informed consent before study entry.

Participants completed a brief descriptive questionnaire asking about their age, sex, marital status, educational achievement, household living status, and self-rated health. A Kardia Mobile 6L electrocardiogram (Kardia; Fremont, CA, US) was used to determine the presence of a heart arrhythmia. Patients were asked to sit quietly for 30 seconds while the electrocardiogram measurement occurred. Algorithms within the Kardia mobile application for determining the presence of an arrhythmia (e.g., atrial fibrillation, bradycardia, tachycardia) were used for determining any arrhythmia presence.

A Jamar hydraulic handgrip dynamometer (Performance Health Supply; Cedarburg, WI, US) collected handgrip strength. A trained interviewer explained and demonstrated all handgrip strength testing procedures. Participants remained seated with their arm on the rests, elbow at 90°, and wrist in a neutral position. Persons needing additional support were allowed to place their arm on an object as appropriate. After fitting the dynamometer to the hand size of each participant, a practice trial for familiarization was permitted. Beginning on the right hand, participants squeezed the dynamometer with maximal effort, exhaling while squeezing for approximately 3 s, and then released their grasp. Participants completed 2 trials on each hand, alternating between hands. A 1-min rest period was allowed between measurements, including if only a single hand is available for testing [21]. The highest recorded handgrip strength was included. Males and females with handgrip strength < 26 kg and < 16 kg were considered as having weakness, respectively [22].

Each participant completed the Mini-Cog quick screening for cognitive impairment with a trained interviewer. The Mini-Cog is a recognized screening tool for assessing cognitive function, which includes a paper-and-pencil based administration involving items such as 3-word recall and clock face drawing [23]. Scores on the Mini-Cog range from 0–5 with higher scores indicating greater cognitive function. Participants with scores < 4 were classified as having a cognitive impairment [23].

Those with a positive screening test for a heart arrhythmia, weakness, or cognitive impairment were advised to visit their primary healthcare provider about their positive screening for additional testing. An interviewer followed-up with participants approximately 3-months later in-person at the pharmacy or over the telephone to determine if they had visited their healthcare provider specifically for the positive screening test performed at the community pharmacy.

All analyses were performed with SAS 9.4 software (SAS Institute; Cary, NC, US). The descriptive characteristics of the participants were presented as mean ± standard deviation for continuous variables and frequency (percentage) for categorical variables for the overall sample and by referral status. An independent t-test analyzed the differences in age by referral status, while chi-squared tests examined differences in sex, marital status, educational achievement, household living status, and self-rated health by referral status. Reasons for referral status and follow-up to determine if a healthcare provider visit from the screenings was completed were presented as frequency (percentage). An alpha level of 0.05 was used for all analyses.

## RESULTS

Table 1 presents the descriptive characteristics of the participants. Overall, participants were aged 74.8 ± 6.4 years and were mostly female (57.5%). Participants recommended for a referral were older (77.4 ± 6.4 years) compared to non-referrals (71.1 ± 11.1 years; p < 0.001). Of the 106 participants included, a total of 51 participants had a positive screening for any assessment, with 9 (17.67%) specifically screening positive for a heart arrhythmia, 24 (47.1%) screening positive for weakness, and 31 (60.8%) screening positive for cognitive impairment. At follow-up, 12 (23.5%) participants reported visiting their primary healthcare provider about the positive screening conducted at the community pharmacy (4 (7.8%) for arrhythmia; 5 (9.8%) for weakness; 4 (7.8%) for cognitive impairment), while 4 (7.8%) total participants were lost at follow-up.

## DISCUSSION

Our pilot study showed promise for initiating participant follow-up with primary healthcare providers regarding positive heart arrhythmia, weakness, and cognitive impairment screenings performed at community pharmacies for older adults. Conducting such screenings may reengage patients with their primary healthcare providers, and support the identification of unknown conditions. While several factors may explain why approximately a quarter of participants followed-up with their healthcare provider about their positive screening in our study, additional patient care communications between pharmacists and physicians may support uptake [24].

Performing health-related screenings during routine visits to other non-physician healthcare providers has been observed. For example, conducting blood pressure assessments during visits to the dentist have demonstrated effectiveness in identifying unknown cases of possible hypertension [25]. Given the frequency of visitation to community pharmacies [8], implementing screenings into routine workflow where possible at such pharmacies may not only uncover undiagnosed cases of age-related health conditions, but also help in establishing a continued coordination of care between patients, pharmacists, and primary care physicians [26].

The integrated care for older people approach (ICOPE) provides a continuum of integrated care that supports patient-centered and coordinated care for health and social domains, including optimizing intrinsic capacity and functional ability during aging [27]. Tools and pathways for promoting healthy aging such as the INSPIRE ICOPE-CARE program presents larger scale clinical practice and public health implementation options [28]. Previous work that conducted larger-scale implementation and feasibility of the INSPIRE ICOPE-CARE guidelines in clinical practice showed a high number of participants were included, with similarly high follow-up rates, suggesting large-scale implementation of ICOPE in clinical practice is feasible [29]. Given that our study includes measures that align with ICOPE’s domains, implementing measures for such domains in different healthcare settings when applicable may further increase scalability.

Our implementation-oriented pilot study was performed in a community pharmacy located inside a frequently visited clinic. Pharmacists are regarded as trusted healthcare professionals, and patients might be interested in expanded services with their pharmacist [30]. Patient preferences may factor into the frequency of community pharmacy visitation and selection, whereby patients generally value patient-pharmacist relationships, costs, convenience, and wait times [31]. Community pharmacies that have established relationships with their patients may also have higher engagement with respect to patient screenings and healthcare provider follow-ups. Other factors such as geographic location of a community pharmacy (urban, rural) may likewise influence implementation of services.

Although community pharmacists have often lacked access to electronic medical records, patient health information exchange platform access has improved a pharmacist’s ability to provide enhanced patient care recommendations when appropriate [32]. However, barriers may limit health information exchange, and fragmentation across platforms may exist. For example, barriers to integrating pharmacists into primary healthcare included regulatory concerns, financial constraints, workforce shortages, limited infrastructure, restricted health information access, and poor collaboration [33]. Policies and payment for service methods to support sustainable and consistent healthcare between pharmacists and other healthcare providers will help to provide a model that could be scaled internationally.

We acknowledge limitations of our implementation-oriented pilot study. Specifically, other screening tests can be performed for arrhythmias and cognitive function, and likewise, other markers may have relevance to older populations in pharmacy settings (e.g., hypertension). Convenience sampling was present for our pilot study for feasibility, which may limit generalizability. Given that this implementation-oriented pilot study was only performed at a single community pharmacy located in a clinic that serves several older adults annually, validity is threatened with factors such as setting (urban vs. rural), pharmacy type (chain vs. independent), and site (clinic vs. non-clinic). Indeterminate/unclassified arrhythmia measures being classified as non-referrals (n = 14) may have underestimated our findings. Follow-up visits were self-reported, which may introduce recall and social desirability biases. Reasons for not seeking follow-up from referral were not collected. Measures included in our study such as the electrocardiogram can be used for other purposes in pharmacy settings [34]. Future research may focus on additional pharmacy specific screenings that are centered on medications including those that might be potentially inappropriate [35], adherence to medications, and examining the impacts on trending medications such as glucagon-like peptide-1 receptor agonists. Consistently leveraging shared patient care resources and communications such as electronic health records and clinical confirmations will help in verifying subsets of referral confirmation when feasible. Moreover, scaling implementation to multiple community pharmacies, closely evaluating feasibility, strengthening measures (e.g., longer follow-ups, repeated measures, verified outcomes), and acknowledging the relevance of measures for pharmacies and patients are practical next steps to supporting enhanced patient care in community pharmacies.

## CONCLUSIONS

This implementation-oriented pilot study found promise for performing arrhythmia, weakness, and cognitive function screenings at a community pharmacy. Given that older adults may frequently visit their community pharmacy each year, these sites of healthcare can provide screenings and education for connecting older adults with their healthcare provider. As such, unknown conditions can be identified and treated as appropriate. Future research should continue examining the role of community pharmacies for different healthcare services.

## Figures and Tables

**Table 1.** Descriptive Characteristics of the Participants.

Variable	Overall (n = 106)	No-Referral Recommended (n = 55)	Referral Recommended (n = 51)
Age (years)	74.8 ± 6.4	71.1 ± 11.1	77.4 ± 6.4*
Female (n (%))	60 (56.6)	36 (65.4)	24 (47.0)
Married (n (%))	66 (62.2)	36 (65.4)	30 (58.8)
Completed Bachelor’s Degree or Higher (n (%))	49 (46.2)	26 (47.2)	23 (45.1)
Lives Alone (n (%))	37 (34.9)	18 (32.7)	19 (37.2)
Excellent or Very Good Self-Rated Health (n (%))	53 (50.0)	26 (47.2)	27 (52.9)

* p < 0.05.

## Data Availability

The dataset from the study is not available because of privacy restrictions.
